# Differential Roles of Hyperglycemia and Hypoinsulinemia in Diabetes Induced Retinal Cell Death: Evidence for Retinal Insulin Resistance

**DOI:** 10.1371/journal.pone.0026498

**Published:** 2011-10-26

**Authors:** Patrice E. Fort, Mandy K. Losiewicz, Chad E. N. Reiter, Ravi S. J. Singh, Makoto Nakamura, Steven F. Abcouwer, Alistair J. Barber, Thomas W. Gardner

**Affiliations:** 1 Kellogg Eye Center, University of Michigan, Ophthalmology and Visual Sciences Department, Ann Arbor, Michigan, United States of America; 2 Department of Ophthalmology, Penn State College of Medicine, Hershey, Pennsylvania, United States of America; The University of Hong Kong, Hong Kong

## Abstract

Diabetes pathology derives from the combination of hyperglycemia and hypoinsulinemia or insulin resistance leading to diabetic complications including diabetic neuropathy, nephropathy and retinopathy. Diabetic retinopathy is characterized by numerous retinal defects affecting the vasculature and the neuro-retina, but the relative contributions of the loss of retinal insulin signaling and hyperglycemia have never been directly compared. In this study we tested the hypothesis that increased retinal insulin signaling and glycemic normalization would exert differential effects on retinal cell survival and retinal physiology during diabetes. We have demonstrated in this study that both subconjunctival insulin administration and systemic glycemic reduction using the sodium-glucose linked transporter inhibitor phloridzin affected the regulation of retinal cell survival in diabetic rats. Both treatments partially restored the retinal insulin signaling without increasing plasma insulin levels. Retinal transcriptomic and histological analysis also clearly demonstrated that local administration of insulin and systemic glycemia normalization use different pathways to counteract the effects of diabetes on the retina. While local insulin primarily affected inflammation-associated pathways, systemic glycemic control affected pathways involved in the regulation of cell signaling and metabolism. These results suggest that hyperglycemia induces resistance to growth factor action in the retina and clearly demonstrate that both restoration of glycemic control and retinal insulin signaling can act through different pathways to both normalize diabetes-induced retinal abnormality and prevent vision loss.

## Introduction

Type 1 diabetes is characterized by multiple metabolic changes, notably insulin deficiency and hyperglycemia. Systemic insulin administration both restores depressed insulin signaling through increased ligand availability, and normalizes blood glucose levels through increased glucose uptake, storage and utilization in insulin sensitive tissues. There has been increasing interest in dissecting the relative contributions of hyperglycemia and hypoinsulinemia to the pathophysiology of diabetes and diabetes complications because current treatments for diabetes fail to normalize metabolism or eliminate the risk of complications, and hypoglycemia limits the use of intensive insulin therapy [1].

A strategy to differentiate the roles of hyperglycemia and insulin deficiency to diabetic complications is to separately restore insulin signaling in an affected tissue without effecting glucose exposure and to correct systemic hyperglycemia without increasing insulin levels. Subconjunctival injection of insulin can accomplish the former in retinal tissue, while systemic treatment with phloridzin, a natural compound extracted from the bark of fruit trees, can accomplich the latter. Phloridzin inhibits the sodium-linked glucose transporters SGLT1 and SGLT2 in kidney tubular and small intestinal epithelium [2]. Daily administration of phloridzin in diabetic animals restores glycemic levels close to normal by increasing renal glucose uptake and thus increased glucose excretion, leading to decreased blood glucose levels. Administration of phloridzin to partially pancreatectomized diabetic rats normalized plasma glucose levels without exogenous insulin administration or raising plasma insulin concentrations [3]. Restoration of normoglycemia with phloridzin improved whole-body insulin sensitivity [4], [5] and residual pancreatic beta-cell function in rodents [6]. Systemic phloridzin and intracerebral insulin administration were recently used to demonstrate that decreased local insulin signaling was the main cause of decreased brain cholesterol biosynthesis in diabetic mice [7]. Renal function was demonstrated to remain intact in humans with primary renal glycosuria in whom glucose reabsorption is totally blocked [8], and phloridzin has been used in numerous studies without reports of deleterious effects on animal behavior or activity, while efficiently normalizing systemic glycemic levels in diabetic animals [7], [9], [10]. SGLT inhibitors are also under evaluation in multiple Phase I and Phase III clinical trials without reports of major side-effects (ClinicalTrials.gov) [11].

Whereas hyperglycemia is often thought to be the primary driver of complications progression, there is reason to suspect that impaired insulin signaling could contribute to the retinal pathology observed in diabetic retinopathy. We and others have shown that diabetes depresses retinal insulin signaling with decreased kinase activities of the insulin receptor and downstream signaling proteins including Akt1 and Akt3 [12], [13], [14]. Insulin receptor and Akt signaling regulate retinal neuronal cell survival in culture [15] and in type 2 diabetic rats [16]. Insulin-deficient diabetes increases retinal cell death within 4 weeks after diabetes onset in rats and increased neuronal apoptosis has been documented in post-mortem human eyes [17], [18]. Therefore, we hypothesized that restoration of retinal insulin signaling and glycemic normalization would exert differential effects on retinal cell survival and retinal physiology during diabetes. Interestingly, both insulin-independent normalization of systemic hyperglycemia and ocular delivery of insulin without normalization of blood glucose partially reversed diabetes-induced retinal cell death. Further analysis demonstrated that both normalization of hyperglycemia and increased ocular insulin signaling reversed diabetes-induced insulin receptor/Akt signaling defects. In keeping with these observations, transcriptomic analysis demonstrated that these treatments normalized partially overlapping sets of retinal genes that were altered by diabetes. Thus, loss of local insulin signaling and systemic hyperglycemia have both common and separable effects on the retina. These findings are important for understanding the contributions of factors in the diabetic milieu that contribute to diabetic retinopathy and may be modifiable to improve visual outcomes in patients.

## Methods

### Ethics statement

All experiments were conducted in accordance with the Association for Research in Vision and Ophthalmology Resolution on the Care and Use of Laboratory Animals and these studies were specifically approved by the Penn State University (IACUC #2008-038 and 2009-095) and University of Michigan (UCUCA #10463) animal care and use committees.

### Induction of diabetes and insulin therapies

Age-matched male Sprague-Dawley rats (Charles River, MA) were housed under a 12 h light/dark cycle with free access to a standard rat chow and water. Diabetes was induced by intraperitoneal injection of streptozotocin (STZ) (65 mg/kg; Sigma, St. Louis, MO) dissolved in sodium citrate buffer, pH 4.5, and control rats received equivalent volumes of buffer alone as described previously [18]. STZ-injected rats were considered diabetic when exhibiting blood glucose levels >13.9 mmol/l (250 mg/dl) within 5 days after diabetes induction (One-Touch meter; Lifescan, Milpitas, CA).

**Figure 1:**
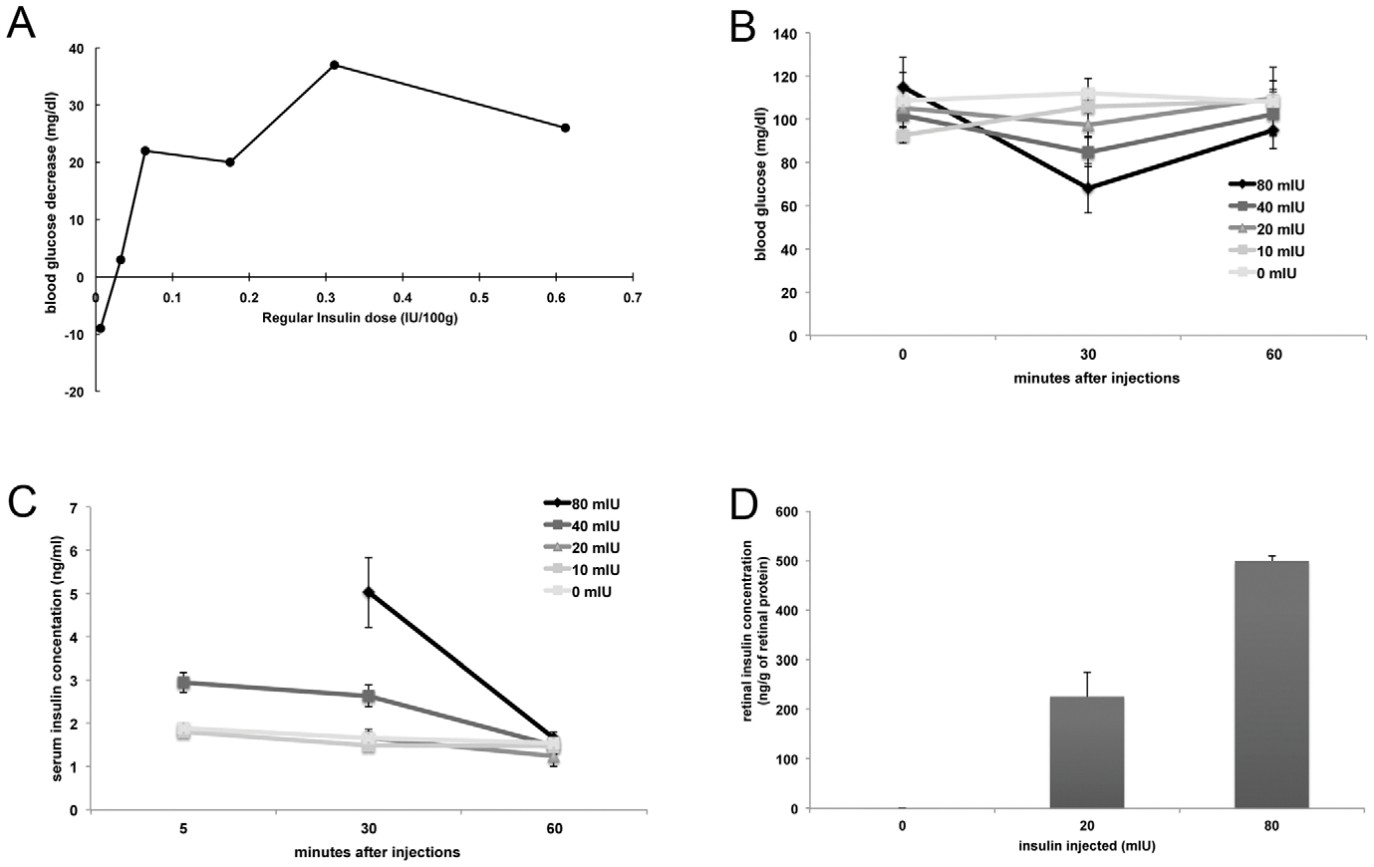
Subconjunctivally delivered insulin reaches the retina without having systemic effects. A dose-response study of the blood glucose levels of non-diabetic control rats 30 minutes after subconjunctival administration of insulin was conducted and showed that doses over 0.0325 IU per 100 g of body weight significantly decreased blood glucose levels (A). Further analysis of lower doses of insulin showed that administration of 20–40mIU of insulin had no systemic effect on serum glucose (B) or insulin (C) levels, while significantly increasing retinal insulin levels (D).

Acute, short-term insulin therapy consisted of a daily subconjunctival injection of Novolin (20 mIU) or the control vehicle (PBS with 0.1% BSA) for the last 4 days of the studies. This dose was chosen on the basis of preliminary studies showing activation of the retinal insulin receptor signaling pathway without reducing blood glucose levels (Figure 1A). The 4 and 12 weeks diabetes duration studies were chosen because they lead to increased neuronal cell death, microvascular leakage, astrocyte defects, microglial cell activation, and impaired insulin receptor signaling [18], [19], [20], [21], [22]. Insulin-independent glycemic control was achieved by phloridzin therapy. The rats received 2 daily sub-cutaneous injections of phloridzin (200 mg/kg of body weight) or vehicle alone (60% 1, 2-Propanediol) during the last 3 full days of the experiment, as well as the morning of the 4th day, 3 h prior to euthanasia. Blood glucose and insulin levels were monitored to determine the efficacy of the treatment while food intake and activity were monitored to insure that the treatments were not appreciably affecting overall wellness. Vitreous glucose was measured at time of harvest to monitor the effect of the therapeutic treatments on ocular glucose levels using a glucometer as previously described by Kirwin et al. [23].

For rapid dissection of retinas, rats were anesthetized with injection of sodium pentobarbital, 100 mg/kg i.p., and killed by decapitation following motor reflex loss. Retinas were immediately frozen in liquid nitrogen and stored at −80°C until analysis (see below).

### Insulin assays

Serum and retinal insulin levels were measured by 2 different methods: the sensitive rat RIA kit (SRI-13K Milipore/Linco), and a Rat/Mouse insulin ELISA kit (EZRMI-13K, Millipore). For both methods serum was obtained by promptly centrifuging the clotted blood at 2,000× g for 15 minutes at 4±2°C. For the RIA, each retina was sonicated in 120 µl of assay buffer supplied in kit with protease inhibitor (Roche), and lysates were rocked at 4°C for 15 minutes followed by centrifugation at 10,000× g for 10 min. The supernatant was used for the RIA as per the manufacturer's protocol. Protein concentrations of tissue lysates were determined using a DC protein assay kit (Bio-Rad, Hercules, CA) and retinal insulin levels were normalized to total retinal protein concentration. For the ELISA, each retina was sonicated in 40 µl of lysis buffer (10 mM HEPES, 42 mM KCl, 5 mM MgCl2, 0.1 mM EDTA, 50 mM sodium pyrophosphate, 1 mM DTT, 1 mM PMSF, 1 mM Na3VO4, 10 mM NaF, 10 mM benzamidine, 10% glycerol, 1% Nonidet P-40, and one protease inhibitor tablet/10 ml) before being centrifuged for 10 min at 10,000× g at 4°C. The ELISA was performed as per the manufacturer's instructions using the protocol option A for serum, with 10 µl of serum and 10 µl of buffer, and option B for the retinal assay, using 20 µl of lysate.

### Cell death detection assays: ELISA and TUNEL

Apoptosis was measured by two complementary approaches previously demonstrated to show corresponding results [24]. A Cell Death Detection ELISA (Roche Diagnostics) was used according to the manufacturer's instructions with minor modifications. Briefly, after homogenizing the retinal tissue, samples were incubated for 30 minutes and centrifuged at 10000× g for 10 min prior to 20 µl of the supernatant, as well as of the positive and negative controls being transferred into the ELISA plate along with the immunoreagent complex. Following incubation and washes, the colorimetric solution was added and incubated until the colorimetric reaction developed. After adding the stop solution, the colorimetric signal was measured with a fluorescence plate reader (SpectraMax Gemini EM; Molecular Devices) with excitation at 405 and 490 nm. Cell death was also measured by terminal transferase dUTP nick end labeling (TUNEL) with horseradish peroxidase detection in whole-mount retinas as described previously [18].

### Kinase activity assays

#### Insulin receptor (IR) and insulin-like growth factor-1 (IGF-1R) IGF-1R kinase assays

Retinas were homogenized by sonication in lysis buffer (see above) and 500 µg of tissue lysates were immuno-precipitated using anti-IR or anti-IGF1R antibodies (Santa Cruz), as described previously [13]. After washing the immune complex, its specific kinase activity was assessed using radiolabeled ATP (25 µCi/ml [^32^P]-ATP, Amersham) incorporation onto receptor specific peptides as described previously [13]. After stopping the kinase reaction by brief centrifugation, the supernatant was spotted onto filter papers that then underwent several washes before being analyzed for radioactive counts on a scintillation counter.

#### Akt isoform-specific kinase assays

Akt isoform-specific kinase assays were performed as previously described [25]. Briefly, supernatants (500 µg of protein) of retinal tissue homogenates prepared as for the IR or IGF1R kinase assay were subjected to immunoprecipitation (1 h at 4°C) with 2 µg of anti-Akt-1 or Akt-3 primary antibody (Millipore). After washing the immune complex, its specific kinase activity was assessed using radiolabeled ATP incorporation onto a crosstide peptide substrate (GRPRTSS-FAEG, 30 µM, Millipore) as described above for the IR and IGF1R kinase assays. In all kinase assays equivalent efficiency of the immunoprecipitation was verified by immunoblot analysis of the immune complexes after sufficient decay of the radioactivity.

### RNA isolation, Real-time RT-PCR and Illumina Microarray analysis

Total RNA from retinal tissues was isolated with Tri-Reagent/BCP (Molecular Research Center, Cincinnati, OH) following standard methods and quality and quantity was assessed using the RNA 6000 Nano LabChip with an Agilent 2100 Expert Bioanalyzer (Agilent, Palo Alto, CA). An equal quantity of RNA from each sample was converted to cDNA using the SuperScript First-Strand Synthesis System for RT-PCR (Invitrogen). Quantitative PCR analysis was performed as described previously [26]. Briefly, quantitative PCR was performed using the 7900HT Sequence Detection System (Applied Biosystems, Foster City, CA), 384-well optical plates, and Assay-On-Demand (Applied Biosystems) gene specific primers and probes. ABI SDS 2.2.2 software and the 2-ΔΔCt analysis method were used to quantify relative amounts of product using beta-actin as an endogenous control. Beta-actin levels were determined to be unchanged in an absolute quantification experiment (data not shown). For the Illumina microarray, 750 ng of purified cRNA was prepared for hybridization of Illumina RatRef-12 Expression BeadChips according to the manufacturer's instructions. Briefly, Chips were incubated in a hybridization oven for 20 h at 58°C before being disassembled, washed and Streptavadin-Cy3 stained. Chips were dried and subsequently scanned using a BeadArray Reader and images were imported into GenomeStudio software v2010.1 (Illumina Inc, San Diego, CA). After performing the quality controls, background subtraction and intra-array normalization, GenomeStudio-exported files were imported into GeneSpring GX11.0 software (Agilent Technologies). The array data are MIAME compliant and have been deposited in the ArrayExpress MIAME compliant database (accession # E-MTAB-771).

### Immunohistochemistry

Immunohistochemistry was performed as previously described [22]. Briefly, eyecups were embedded in OCT and snap-frozen in dry ice, cooled with 2-methylbutane, directly after enucleation. Sections (10 µm) from each experimental group were mounted on the same slide. The slides were blocked with donkey serum before incubations at 4°C overnight with the primary antibodies against GFAP (Roche) and Cyanine2 conjugated secondary antibodies were used (Jackson Immunoresearch) and after mounting in Gel/Mount (Biomeda, Foster City, CA) the resulting immunolabeling was examined and photographed using a confocal microscope (Leica, Lasertechnik GmbH). Controls were prepared by omitting the primary antibody during the incubation; no specific staining could be detected in these controls.

### Pathway Analysis

Microarray data were analyzed using the MetaCore™ (Genego Inc.) software suite for pathway analysis to identify the most significant pathways affected by diabetes and reversed by the independent treatments.

### Statistical Analysis

ANOVA models with heterogeneous variances, adjusted for the replication of the experiment, were fit to the data to assess differences between control, diabetic and treated animals. The means ± SEM and statistically significant differences are reported. Analyses were performed using non-repeated measures ANOVA followed by the SNK test for multiple comparisons or *t*-test for a single comparison.

## Results

### Subconjunctival administration of low dose insulin reaches the retina without systemic effects

Systemic insulin treatment allows for relatively good control of diabetes through mechanisms that can be attributed to normalization of the glycemic levels and activation of insulin signaling pathways in various tissues. We previously demonstrated that intravitreal injection of insulin restores retinal IR activity [14]. To differentiate between the two effects, we first performed a dose-response study of subconjunctivally administered insulin followed by analysis of glucose and insulin blood levels. Preliminary analysis showed that blood glucose levels of non-diabetic control rats were similarly decreased by subconjunctival insulin doses greater than 50 mIU/100 g of rat body weight (Figure 1A). We then further analyzed the effects of doses below this threshold on blood insulin and glucose levels as well as retinal insulin concentration. Subconjunctival injections of 50 mIU insulin lowered blood glucose level at 30 min after injection, whereas lower doses up to 40 mIU had no significant impact on blood glucose levels at 30 and 60 min after injection (Figure 1B). While the 80 mIU dose raised serum insulin levels approximately 3 fold, the 20 mIU had no effect (Figure 1C). In contrast, subconjunctival injection of the same 20 mIU dose, significantly increased insulin content within retinal tissue (Figure 1D). Furthermore, insulin administered by subconjunctival injection was detected in the retinas as soon as 5 minutes after injection and was detectable for over an hour after injection (data not shown). Therefore, the doses of subconjunctival insulin used for subsequent studies are specific to the eye and do not alter systemic metabolism.

### Subconjunctival insulin administration specifically increases retinal insulin signaling in diabetic rats while phloridzin normalizes hyperglycemia in an insulin-independent manner

**Figure 2:**
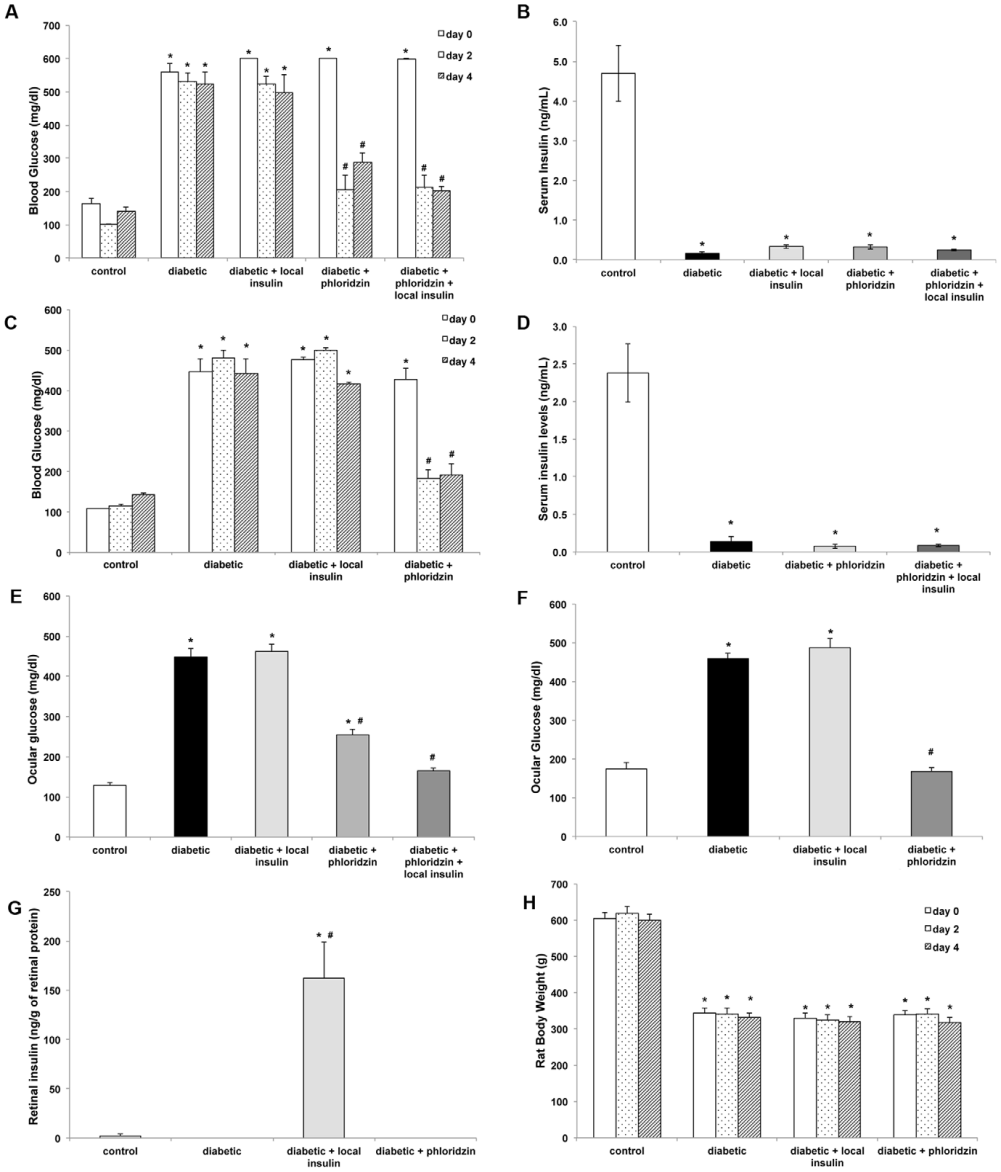
Distinct physiological effects of subconjunctivally delivered insulin and systemic phloridzin treatment. Two or four days of local insulin administration (20 mIU) had no effect on serum glucose (A, C) or insulin (B, D) levels of 4 (A–B) or 12 (C–D) weeks diabetic animals, while phloridzin administration rapidly normalized serum glucose levels at both 4 (A) and 12 (C) weeks of diabetes without affecting serum insulin levels (B, D). Ocular insulin administration had no effect on vitreous glucose levels (E, F) while greatly increasing retinal insulin levels (G) whereas phloridzin reduced high vitreous glucose levels (E, F) without affecting retinal insulin levels (G). Rat body weights were unaffected irrespective of the treatments as demonstrated in 12 wks diabetic STZ-rats (H). n≥8/group; *significantly different from control the same day (*P*<0.05); ^#^significantly different from untreated diabetic the same day (*P*<0.05).

**Figure 3:**
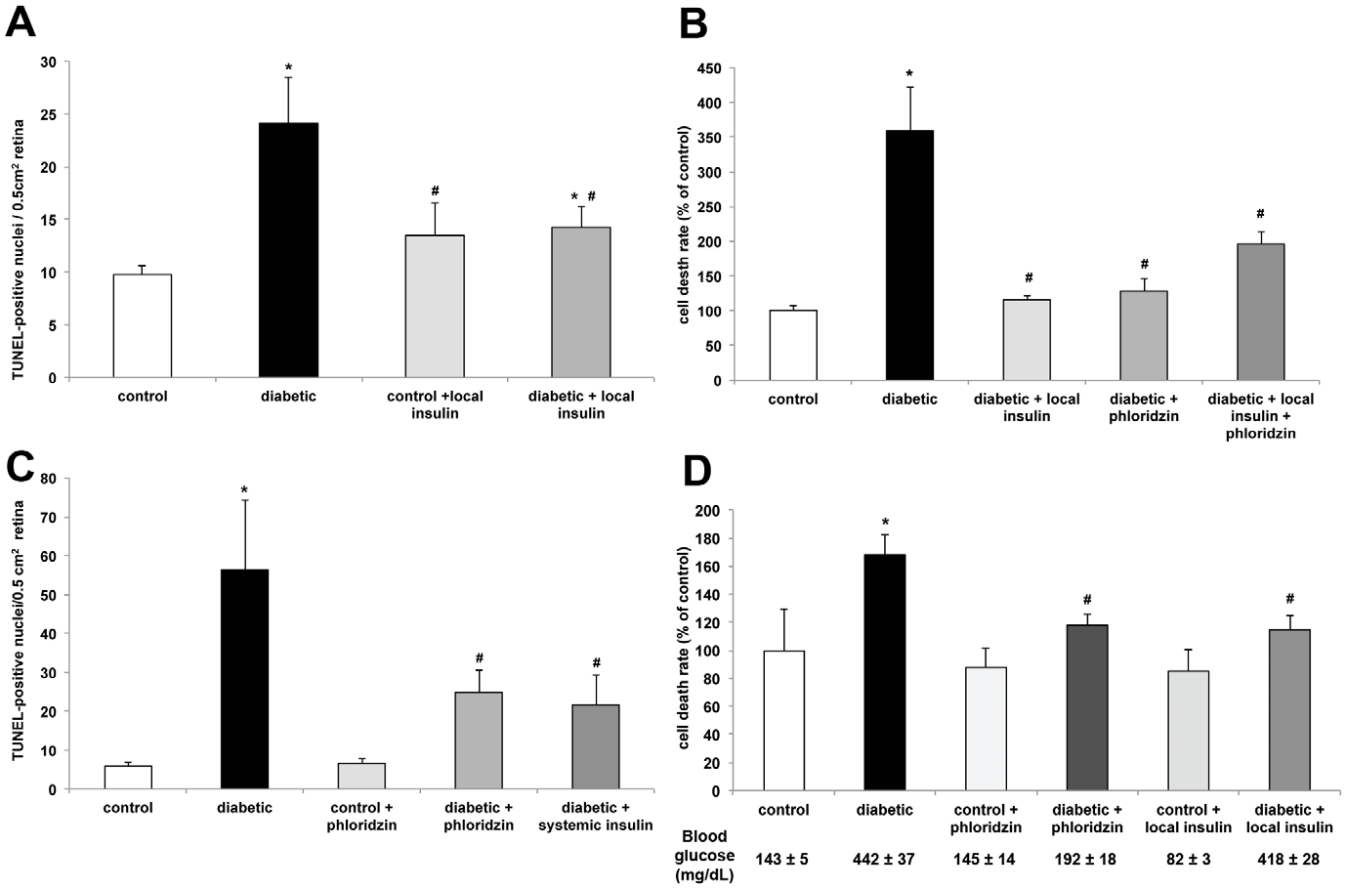
Ocular insulin administration and serum glucose normalization can both block diabetes induced retinal cell death. Retinal cell death was measured in diabetic rats (4 and 12 weeks duration) following local or systemic insulin administration and/or phloridzin treatment for 4 days using TUNEL staining or DNA fragmentation ELISA Assay. The number of TUNEL positive cells in 4 weeks diabetic rat retinas was significantly reduced by local insulin administration (A) while cell death rate analysis using a DNA fragmentation ELISA assay showed a significant reduction by either local insulin administration or phloridzin treatment without additive effect when the treatments were combined (B). Similarly, the number of TUNEL positive cells in 12 weeks diabetic rat retinas was significantly reduced by phloridzin and systemic insulin treatment (C) while cell death rate measured using the DNA fragmentation assay was also reduced by phloridzin and local insulin administration (D). n≥6/group; *significantly different from control (*P*<0.05); ^#^significantly different from diabetic (*P*<0.05).

The systemic effects of both local insulin and phloridzin treatments were analyzed by monitoring glucose and insulin serum levels. Daily subconjunctival administration of 20 mIU of insulin for 4 consecutive days to rats with 4 weeks of STZ-induced diabetes did not reduce the hyperglycemia (Figure 2A). Similarly, no differences in serum insulin levels were detected between the treated and untreated diabetic rats, demonstrating that subconjunctival administration of low doses of insulin had no effect on the systemic hypoinsulinemic condition (Figure 2B). Phloridzin administration significantly reduced mean serum glucose levels of diabetic rats from 531 mg/dL and maintained them at normal levels (206 mg/dL) for at least 6 h post-injection at day 2 (Figure 2A). Phloridzin had no effect on serum insulin levels confirming that the effect of phloridzin on serum glucose levels is independent of plasma insulin (Figure 2B). Specificity of the treatments was also observed when rats with longer duration of diabetes (12 weeks) were treated (Figure 2C and 2D). Ocular effects of the treatments were also assessed by monitoring vitreous glucose and retinal insulin levels at the end of the treatment. In correlation with the systemic results, the mean vitreous glucose concentrations were reduced from means of 448 to 254 mg/dL by phloridzin treatment, but were unaffected by local insulin administration (Figure 2E and 2F), and retinal insulin levels did not increase in phloridzin treated rats (Figure 2G). In contrast, subconjunctival injection of insulin in diabetic rats lead to significantly increased retinal insulin levels (Figure 2G). Neither local insulin administration nor systemic phloridzin treatment significantly affected the body weights or food consumption of rats during the 4 days of treatment (Figure 2H). Also, while retinal insulin levels after subconjunctival injections are supra-physiologic, they did not cause any increase in retinal cell death (Figure 3). Together, these data demonstrate that the ocular metabolic parameters were affected in specific manners by these pharmacological manipulations.

### Increased ocular insulin signaling and systemic blood glucose reduction protect retinal cells from diabetes-induced cell death

We have previously demonstrated that diabetes significantly increases retinal cell death within 4 weeks after the onset of hyperglycemia, systemic insulin administration restores depressed insulin receptor signaling and cell death, and intravitreal insulin injection restores retinal insulin receptor activity [14]. In the present study, subconjunctival administration of low-dose insulin partially prevented this increased cell death as demonstrated by both reduction in the number of TUNEL-positive retinal cells and DNA fragmentation in the treated rats when compared to the untreated diabetic animals (Figure 3A and 3B). Both subconjunctival administration of low-dose insulin and systemic administration of phloridzin reversed the increased retinal cell death observed in 4 weeks diabetic rats (Figure 3B). Interestingly, applications of both treatments reversed diabetes-induced retinal cell death to similar degrees (over 60%) after longer duration of diabetes as demonstrated by the effect on both the number of TUNEL positive cells (Figure 3C) and DNA fragmentation (Figure 3D) in retina from 12 weeks diabetic animals. Since the two treatments have different effects on hyperglycemia and insulin levels, they were then used in combination to test for a potential additive effect. Surprisingly, no additive effect was observed, suggesting that both treatments use similar or converging parallel intracellular signaling pathways that are individually sufficient, at least for short-term cell survival.

### Subconjunctival insulin administration and phloridzin treatment both partially restore diabetic-induced retinal insulin signaling defects

**Figure 4:**
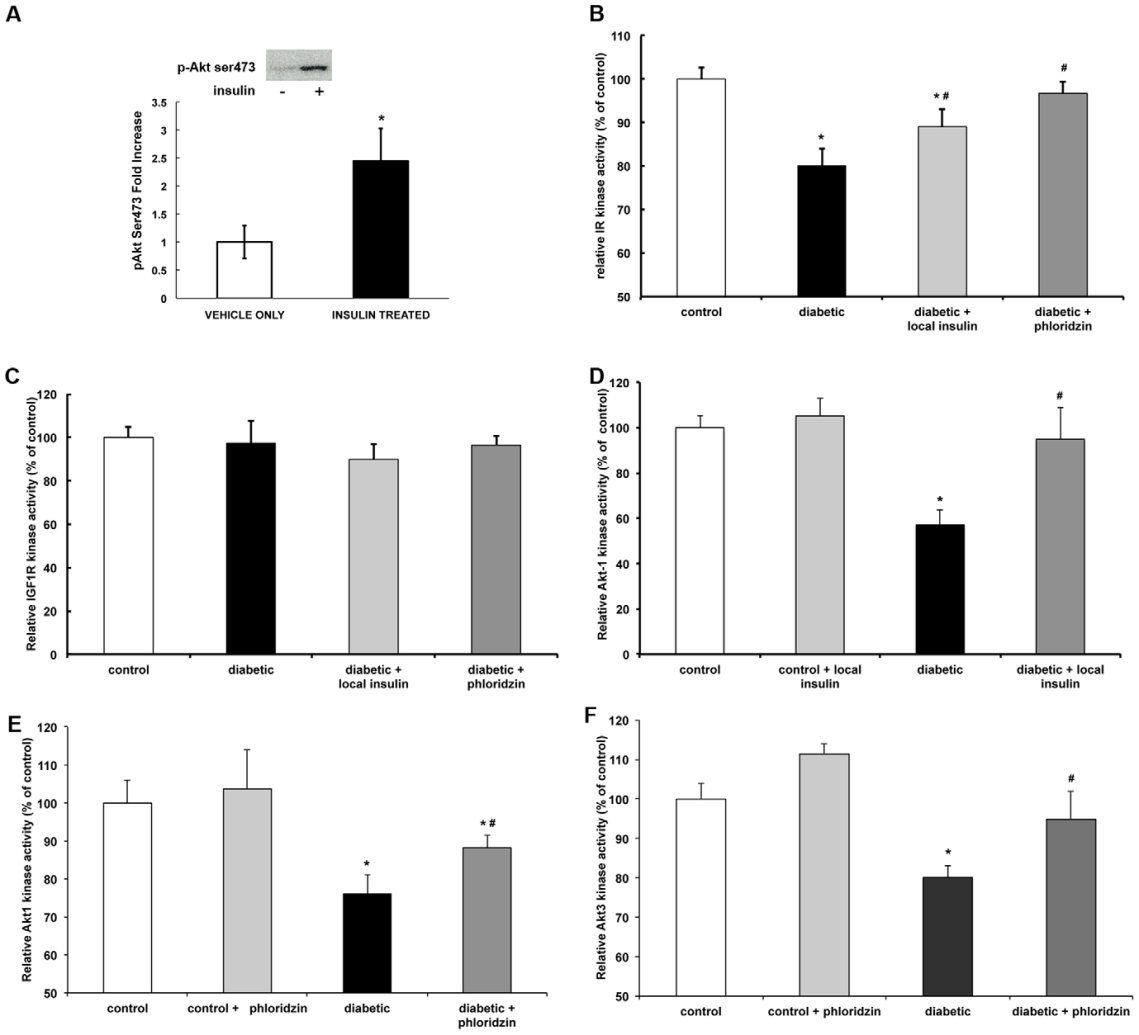
Ocular insulin administration and phloridzin partially restore local insulin signaling. Representative immunoblots and quantification of Akt phosphorylation in retinas of non-diabetic rats receiving subconjunctival insulin. After 30 min, retinas were analyzed for Akt phosphorylation. Retinal Akt serine 473 phosphorylation was significantly elevated in eyes receiving insulin (*p<0.05; A). The effect of local insulin and phloridzin treatment on the insulin signaling pathway was measured using kinase activity assays for IR (B), IGF1R (C), Akt1 (D–E) and Akt3 (F). Both local insulin and phloridzin treatment restored the diabetic induced IR activity after 12 weeks of diabetes (B) without any effect on the IGF1R activity (C). Activity of the downstream kinase Akt1 was also restored by both local insulin and phloridzin treatment (D–E) while Akt3 activity was restored by phloridzin treatment. n≥8/group; *significantly different from control (*P*<0.05); ^#^significantly different from diabetic (*P*<0.05).

Next, we examined the mechanisms by which subconjunctival injection of insulin and systemic phloridzin treatment prevented the death of retinal cells. First, we demonstrated that the increased retinal insulin levels after subconjunctival injection of insulin in control rats correlated with increased Akt phosphorylation when compared to basal levels, indicating the activation of the insulin signaling pathway and confirming the biological activity of the exogenous insulin reaching the retina (Figure 4A). We then studied the effects of both treatments on the insulin signaling pathway and its disruption during diabetes. Reduction of the insulin receptor activity induced by diabetes was partially reversed by both ocular insulin administration and phloridzin treatment (Figure 4B). As previously reported, neither diabetes nor any of the treatments had any impact on retinal IGF-1 receptor activity (Figure 4C). Since Akt isoforms are key downstream kinases of the insulin signaling cascade and crucial elements in retinal cell survival [17], we also analyzed their activity in response to diabetes and both treatments. As previously demonstrated, Akt1 and Akt3 activities in the retina were reduced by diabetes (Figure 4D and 4F). Local insulin treatment totally restored Akt1 activity (Figure 4D), while phloridzin treatment partially restored both Akt1 and Akt3 activities (Figure 4E and 4F). This finding suggests that both local insulin administration and blood glucose normalization can restore retinal insulin receptor signaling, although direct activation by the ligand is more effective.

### Increased local insulin and systemic normalization of glycemia protect retinal neurons through different pathways

In addition to targeted studies of the insulin receptor signaling pathway, we also employed a discovery approach to examine the genomic effects of insulin administration and glucose reduction. A microarray analysis of the retinal transcriptome changes of diabetic rats and diabetic rats that received local insulin or phloridzin treatments revealed 983 genes were significantly affected greater than 1.2 fold (statistically significant with p<0.05) by diabetes (Table S1). Of those, close to 700 were at least partially (30% or more when compared to untreated diabetic) reversed by at least 1 of the 2 treatments. More than 600 of these genes were reversed by subconjunctival insulin administration, while only 298 were reversed by phloridzin treatment. Interestingly over 200 of them were reversed by both treatment, but only a third of them were reversed to a similar extent. Gene pathway analysis gives critical information on the main biological aspects that are affected by the pathological and treatment conditions, and is critical to understanding of the pathophysiology of other diabetic complications [27]. Interestingly, while over half of the genes reversed by phloridzin were also reversed by local insulin administration, pathway analysis using the MetaCore knowledge database and software (Genego Inc.) clearly showed that local insulin signaling primarily affects inflammatory, cytoskeleton regulatory pathways and angiogenesis related pathways (Table 1), while the effects of phloridzin treatment are mainly on growth factor signaling pathways without decreasing the inflammatory component (Table 2). This analysis strongly demonstrated that local insulin signaling but not hyperglycemia was crucial in controlling several aspects of the immune response to diabetes, including the classical complement pathway, cytokine-induced signaling pathways and lipid metabolism in the retina. Control of hyperglycemia was demonstrated to be more associated with pathways involved in the regulation of cytoskeleton remodeling, cell signaling and energy metabolism.

**Table 1:** Top ten pathways affected by diabetes and reversed by ocular insulin administration.

ranking	Maps	min(pValue)
1	Immune response_Classical complement pathway	4.112E-12
2	Cell adhesion_Role of tetraspanins in the integrin-mediated cell adhesion	5.144E-09
3	Immune response_Lectin induced complement pathway	9.611E-08
4	Regulation of lipid metabolism_Regulation of lipid metabolism via LXR, NF-Y and SREBP	1.089E-07
5	Development_Role of IL-8 in angiogenesis	5.124E-07
6	Cytoskeleton remodeling_Regulation of actin cytoskeleton by Rho GTPases	8.567E-06
7	Immune response_IL-22 signaling pathway	7.801E-05
8	Cytoskeleton remodeling_Neurofilaments	1.972E-04
9	Immune response_IL-5 signalling	4.053E-04
10	Development_Slit-Robo signaling	4.831E-04

List of the top ten pathways obtained considering only the list of genes from the microarray analysis that were affected by diabetes and reversed by local insulin administration using MetaCore™ (Genego Inc.) software. Pathways are ranked based upon p-value.

**Table 2:** Top ten pathways affected by diabetes and reversed by phloridzin treatment.

ranking	Maps	min(pValue)
1	G-protein signaling_RhoB regulation pathway	5.335E-03
2	IL-1 beta-dependent CFTR expression	5.335E-03
3	NGF activation of NF-kB	1.704E-02
4	G-protein signaling_G-Protein alpha-s signaling cascades	2.564E-02
5	Cell adhesion_Role of tetraspanins in the integrin-mediated cell adhesion	2.698E-02
6	dATP/dITP metabolism	2.798E-02
7	Signal transduction_cAMP signaling	2.836E-02
8	Regulation of lipid metabolism_Regulation of lipid metabolism via LXR, NF-Y and SREBP	2.836E-02
9	Translation _Regulation of EIF2 activity	2.976E-02
10	Transcription_NF-kB signaling pathway	2.976E-02

List of the top ten pathways obtained considering only the list of genes from the microarray analysis that were affected by diabetes and reversed by systemic phloridzin treatment using MetaCore™ (Genego Inc.) software. Pathways are ranked based upon p-value.

**Figure 5:**
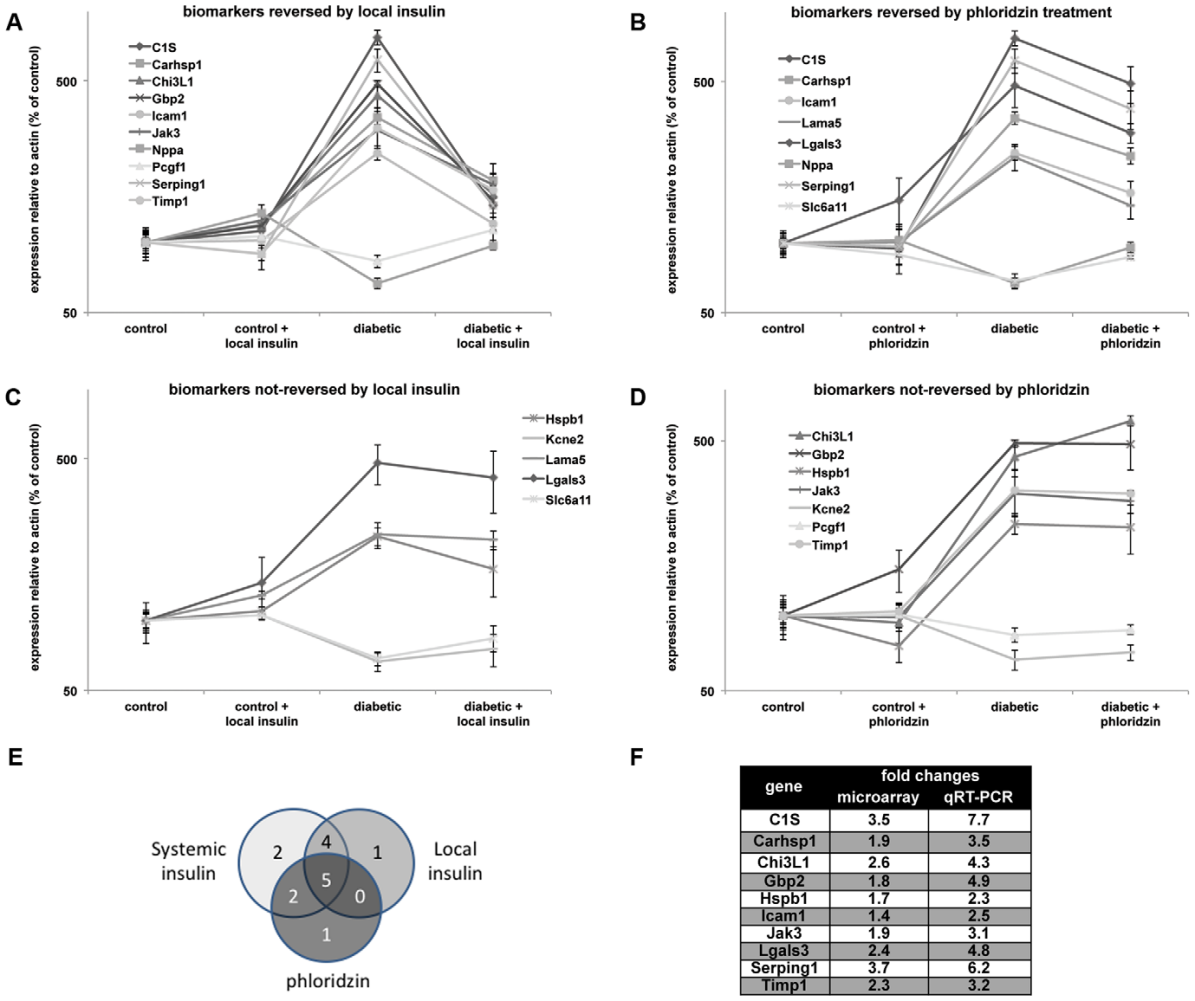
Local insulin administration and phloridzin treatments differentially affect the previously characterized diabetic retinopathy biomarker panel. PCR analysis of the expression of the 15 genes of the diabetic retinopathy biomarker panel was performed on retinal mRNA samples from diabetic rats that received local insulin or phloridzin treatments. Local insulin (A, C) and phloridzin (B, D) respectively normalized the expression of 10 and 8 of those 15 genes. Comparison of the effect of both treatments on the expression of the panel demonstrated that only 5 of the normalized genes were common between the 2 treatments (E) demonstrating that they only partially overlap. Fold changes correlation of 10 of the genes of the biomarker panel detected by microarray and qRT-PCR (F). n≥4/group; *significantly different from control (*P*<0.05); ^#^significantly different from diabetic (*P*<0.05).

Complementary to the microarray analysis, the effects of both treatments on a panel of previously developed mRNA biomarkers of diabetic retinopathy [28] was analyzed and demonstrated that the two treatments differentially affected these biomarkers. Local insulin and phloridzin reversed 10 and 8 of the 15 biomarkers, respectively, but only 5 of these were shared between the two treatments (Figure 5). Also of note, our previous study found that changes in13 of these 15 same mRNA biomarkers were significantly reversed by systemic insulin treatment [28]. Remarkably, local insulin and phloridzin each reversed one of the 2 biomarkers not affected by systemic insulin (Figure 5E), QRT-PCR analysis of this panel of genes also allowed for the validation of the results of the microarray analysis as demonstrated by the good accordance between the levels of expression detected by both methods (Figure 5F).

**Figure 6:**
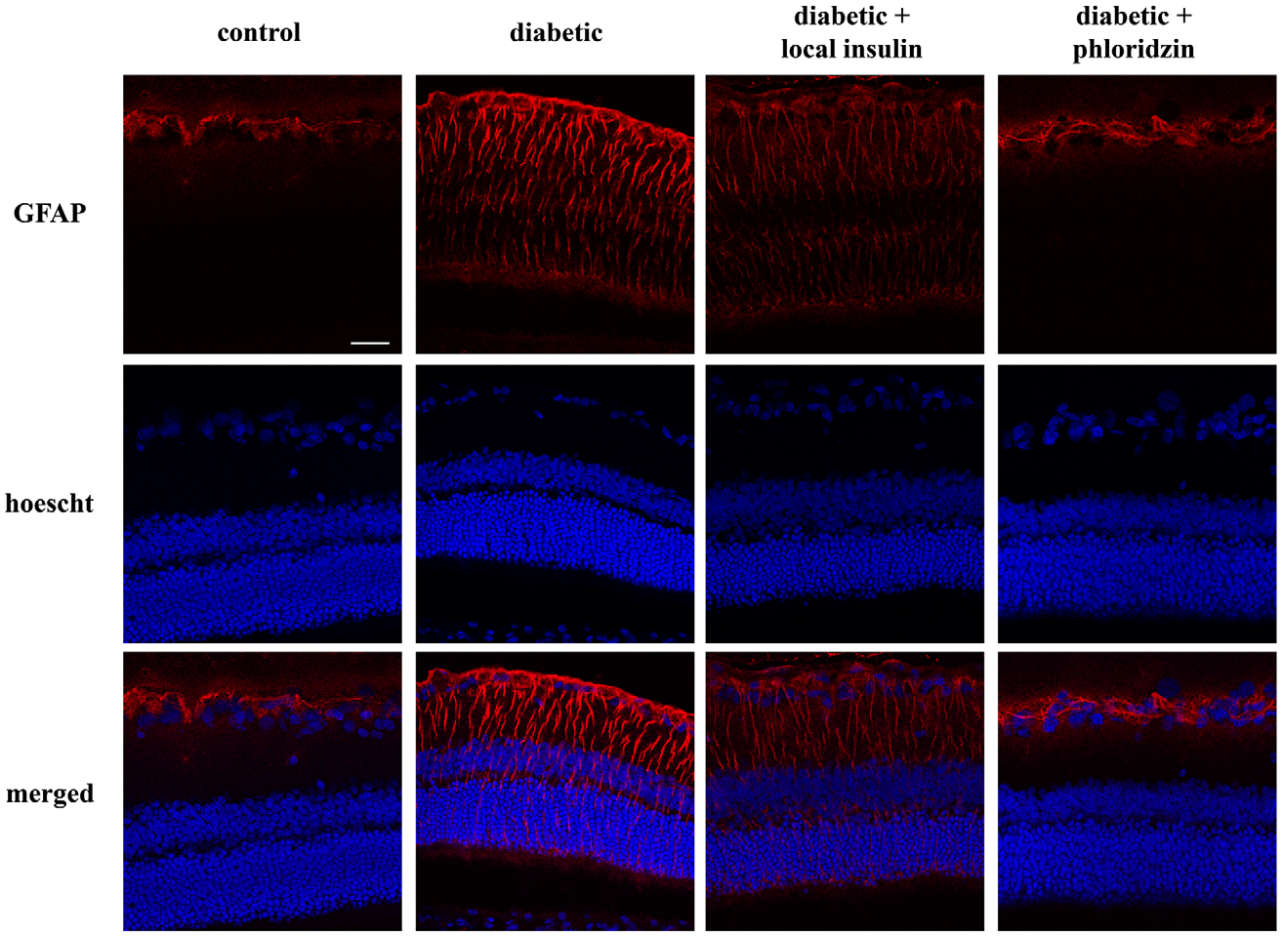
Phloridzin treatment completely reverses the diabetes-induced retinal gliosis while local insulin only reduces it. Immunolocalization of GFAP in normal and 12 week diabetic rats after phloridzin or local insulin treatment. The retinal sections were counterstained with Hoecht to visualize nuclei layers (blue). As previously described, GFAP was only detected in the astrocytes in control animals where it was reduced by diabetes in correlation with an induction in the Müller cells. While local insulin administration only reduced the intensity of the staining in the Müller cells, GFAP could only be detected in astrocytes of phloridzin treated rats. The sections selected are representative of the results observed in 3 independent animals for each conditions.

Pathway analysis of the microarray data strongly suggested differential effects of both treatments. The first two pathways highlighted by the pathway analysis, G-protein signaling and IL-1 beta-dependent CFTR expression, along with the effect on ATP metabolism suggested an impact on retinal glial cells. This prompted us to assess the effect of both treatments on gliosis, as indicated by Müller cell expression of glial fibrillary acid protein (GFAP) in the retinas of diabetic animals. As shown in Figure 6, phloridzin reversed the increased GFAP immunoreactivity in Müller cells from diabetic animals leading to its sole detection in the astrocytes, while local insulin administration only partially decreased the intensity of the gliosis. This observation again confirms the differential mechanisms and responses involved in the beneficial effects of the two treatments.

## Discussion

Diabetic retinopathy is one of the major complications of diabetes but the selective impact of hyperglycemia and hypoinsulinemia on retinal cell response have not been determined *in vivo*. We previously showed that systemic insulin therapy could restore retinal insulin signaling and prevent retinal cell death induced by diabetes [14], [18]. In the present study we employed a double approach using phloridzin and local ocular insulin administration to dissect the effects of decreased retinal insulin signaling and systemic hyperglycemia and their respective roles in the pathophysiology of diabetic retinopathy. This work clearly demonstrates for the first time that hyperglycemia and decreased retinal insulin signaling each exert common and distinct effects on retinal pathophysiology, and that their combined disruptive effects contribute to increased retinal cell death during diabetes. Thus, the retina responds to both tissue insulin availability and nutrient levels, similar to peripheral insulin-sensitive organs. These effects involve post-translational regulation of the retinal insulin receptor signaling pathway and gene expression changes. The restoration of pro-survival pathways by phloridzin treatment along with the partial effect of subconjunctival insulin administration on the same pathways suggest retinal growth factor resistance due, at least in part, to systemic hyperglycemia. These findings are important to the design of therapeutic strategies to minimize hypoglycemia and long-term complications. Targeting increased insulin action in accessible tissues may augment the benefits of systemic insulin therapy.

We first demonstrated that subconjunctival injection of low dose of insulin (20 mIU) once daily for 4 days significantly increased the retinal insulin concentration without affecting serum glucose and insulin levels (Figure 1). We also showed that this exogenous insulin in the retina was biologically active, as indicated by the increase in phosphorylation on the serine 473 residue of Akt (Figure 3), which is one of the kinases downstream of the insulin receptor that mediates neuroprotection [29]. Likewise, we demonstrated that twice daily subcutaneous administration of the SGLT1 and SGLT2 glucose transporter inhibitor phloridzin, restored normal glycemia without affecting systemic or ocular levels of insulin (Figure 2). Rossetti et al. [6] observed increased insulin secretion following phloridzin injections in a partially pancreatectomized rat model, while Lisato et al [5], similarly to us, showed no effect of phloridzin on serum insulin levels in a streptozotocin-induced diabetic rat model. The difference between these studies probably reflects a more profound loss of beta cells in the latter model, preventing any potential stimulation of insulin secretion by remaining beta cells. Hong et al. [4] used phloridzin to examine the insulin-independent effects of glycemic normalization on peripheral and hepatic insulin resistance. A major difference between the brain and the retina and the peripheral insulin-sensitive tissues is the blood-brain and blood-retinal barriers, which control nutrient exchange between the systemic circulation and these tissues. In this study, we used this characteristic to independently study the effects of insulin-independent glycemic normalization and locally-restricted insulin signaling stimulation.

Normalization of blood glucose and increased local retinal insulin levels both reversed the diabetes-induced retinal cell death (Figure 3) in correlation with the reversal of retinal insulin signaling as demonstrated by complete or partial restoration of IR and Akt1 kinase activity (Figure 4). We have shown that retinal insulin receptor signaling is disrupted in insulin-deficient diabetes but it was unclear whether it results from hyperglycemia and/or hypoinsulinemia. In the present study, we show that the hyperglycemia was only partially responsible for the loss of activity of the retinal insulin signaling pathway. The reversal observed when retinal insulin levels were increased suggests that this is also due to loss of ligand or ligand sensitivity. Insulin resistance has been shown in the vasculature of rats with Type 2 diabetes [30] but this is, to the best of our knowledge, the first evidence for impaired insulin action in response to hyperglycemia in the retina. The beneficial effects of local insulin on the viability of sensory neurons in the retina in this study parallel the improved nerve conduction velocity observed in diabetic rats following insulin injection adjacent to the sciatic nerve or intrathecally [31], [32].

Inflammation is increasingly understood as a central component of diabetes and its complications. Our pathway analysis of the effects of phloridzin and local insulin on the retinal transcriptome changes induced by diabetes clearly demonstrated that only local insulin specifically repressed retinal inflammation, particularly the complement activation. Gene expression profiling comparison of isolated Müller cells from diabetic and non-diabetic STZ-rats identified a large cluster of genes associated with inflammation that were highly upregulated in diabetes [33]. Among those genes were several components of the complement pathway and other aspects of inflammation including antigen presentation and cell adhesion, which has also been identified in our analysis as the second most represented pathway specifically, reversed by local insulin administration. Insulin's effect on inflammation could explain in part why local insulin was able to reduce the Müller cell activation observed in diabetes (Figure 6). Activation of the complement pathway has been demonstrated by increased protein expression of several factors such as C3, C4b, C9 and factor B in the vitreous of diabetic patients with proliferative diabetic retinopathy [34]. The same study also showed increased mRNA expression of C3 and factor B in the retina from other diabetic patients with proliferative diabetic retinopathy (PDR). These data suggest a specific potential for ocular insulin in addition to systemic insulin for the treatment of the inflammatory component of diabetes complications.

The retinal lipid metabolism pathway is also specifically restored by local insulin but not phloridzin. Several studies have investigated the specific roles of insulin and hyperglycemia using similar approaches to study their impact on brain metabolism. One study showed that insulin deficiency is the major driver of the defects in the basal hypothalamo-pituitary-adrenal function in diabetes [35] while another study recently used intraventricular insulin versus systemic phloridzin treatment to compare the respective roles of insulin and glycemia in the control of lipid in the brain during diabetes [7]. Suzuki et al. demonstrated that brain insulin deficiency, rather than hyperglycemia, was directly responsible for the reduced cholesterol biosynthesis observed during diabetes. Cellular cholesterol and fatty acid homeostasis are transcriptionally regulated by three members of sterol regulatory element-binding protein (SREBP) family: SREBP-1a, SREBP-1c, and SREBP-2. Of these, SREBP-2 preferentially activates genes responsible for cholesterol synthesis [36]. The authors found brain *Srebf2* and *Srebf1a* decreased in diabetic mice and reversed by systemic insulin. In the retina, we found decreased *Srebf2* while *Srebf1* was increased, neither of which was normalized by phloridzin, whereas local insulin totally reversed *Srebf2* but marginally affected *Srebf1*. In our microarray analysis, local insulin also restored caveolin-1 expression, a protein essential for the transport of cholesterol and sphingolipids. This finding suggests that local insulin signaling may be important for the regulation of retinal cholesterol synthesis and its proper subcellular localization. Our study also suggests that local insulin, which restored SREBP-2 but not SREBP-1 expression, might specifically restore cholesterol synthesis but not perturbations affecting sphingolipids. This could be demonstrated by further studying the effect of local insulin on the reduction of fatty acid elongases observed in rats with untreated short-term diabetes [37]. This close relationship between insulin signaling and lipid metabolism was recently demonstrated by the fact that disruption of plasma membrane lipid rafts with beta-cyclodextrin was associated with IR signaling dysfunction in retinal neurons [38].

Pathway analysis of the specific effects of phloridzin treatment highlighted its impact on the reversal of the deregulation of G-protein signaling. Interestingly, among other genes, phloridzin restored the expression of mDIA2, a protein involved in stress fiber formation. This result prompted us to assess the effect of both treatments on the well-described reactive gliosis observed in the retina during diabetes. We showed that while subconjunctival insulin administration only resulted in a reduction of the intensity of the GFAP staining in Müller cells, phloridzin treatment totally restored the original expression pattern of GFAP with the sole expression in the astrocytes. These data suggests that phloridzin could directly reverse the reactive gliosis induced by diabetes through the control of the expression of mDIA2 and potentially other proteins that regulate cytoskeleton remodeling [39]. Our analysis also showed that phloridzin specifically restored pathways regulated by interleukin-1 beta and nerve growth factor, the receptors for which and themselves are expressed by retinal glial cells [40], [41], [42], once more pointing out the role of Müller cells in the effects of phloridzin on diabetes. We showed that phloridzin but not local insulin administration reduced ocular glucose levels. One of the effects of elevated glucose is increased activation of the hexosamine biosynthesis pathway (HBP), which leads to increased addition of O-GlcNAc modifications which is known to be a mechanism of inhibition of protein action, including the insulin signaling pathway, by competition with activating phosphosites [43]. Our microarray analysis revealed that phloridzin specifically reversed the effect of diabetes on the expression of 2 enzymes involved in the regulation of glucosamine, heparan sulfate (glucosamine) 3-O-sulfotransferase 2 and 5 (respectively Hs3st2 and Hs3st5). The effect of phloridzin on those enzymes could explain the difference between activating the insulin signaling pathway by increasing local insulin levels and restoring the same insulin signaling pathway by normalizing systemic blood glucose using phloridzin. This hypothesis is supported by the fact that systemic insulin similarly restores Hs3st2 expression [44] and could explain why Müller cells remain partially activated (gliosis) despite local insulin-treatment while activation is totally reversed by phloridzin treatment and systemic insulin therapy [45], confirming that local insulin administration could complement systemic insulin treatment.

Diabetes increases the Km of glucose transport systems across the BRB [46] so higher concentrations of glucose in blood are necessary for same amount to enter the retina suggesting a mechanism in the barrier/retina partially counter-acting the increase blood glucose and decreasing the entry of glucose in the retina. Phloridzin is not expected to have a direct retinal effect since *SGLT2* mRNA has not been detected in the retina confirming that the effect observed here is not related to an ocular effect of phloridzin. However, phloridzin also has antioxidant properties and is a better inhibitor of lipid peroxidation than 17-B-estradiol [47], [48]. These properties, which could be involved in the protective role of phloridzin against diabetes in retinal cells will be further investigated in the future.

These findings are clinically relevant because they indicate that both systemic glucose levels and ocular insulin action influence the viability of retinal cells via the actions of pro-survival kinases and the expression of inflammatory mediators and lipid synthetic pathways. Therapeutic strategies that limit nutrient levels and/or augment ocular insulin action may enhance the prognosis for vision in persons with diabetes.

## Supporting Information

Table S1 **Retinal transcriptome changes in response to diabetes and their reversal by ocular insulin administration or systemic phloridzin treatment.**
(XLSX)
